# Treatment of spinal muscular atrophy with Onasemnogene Abeparvovec in Switzerland: a prospective observational case series study

**DOI:** 10.1186/s12883-023-03133-6

**Published:** 2023-02-28

**Authors:** Georg M. Stettner, Oswald Hasselmann, Anne Tscherter, Elea Galiart, David Jacquier, Andrea Klein

**Affiliations:** 1 Neuromuscular Center Zurich and Department of Pediatric Neurology, University Children’s Hospital Zurich, University of Zurich, Steinwiesstrasse 75, CH-8032 Zurich, Switzerland; 2 Department of Neuropediatrics, Children’s Hospital of Eastern Switzerland, St. Gallen, Switzerland; 3 Institute of Social and Preventive Medicine, University of Bern, Bern, Switzerland; 4 Pediatric Neurology and Neurorehabilitation Unit, Lausanne University Hospital, Lausanne, Switzerland; 5 Division of Neuropediatrics, Development and Rehabilitation, Department of Pediatrics, Inselspital, Bern University Hospital, University of Bern, Bern, Switzerland

**Keywords:** Spinal muscular atrophy, Onasemnogene Abeparvovec, Zolgensma, Gene therapy, Switzerland

## Abstract

**Background:**

Spinal muscular atrophy (SMA) is a rare neuromuscular disorder leading to early death in the majority of affected individuals without treatment. Recently, targeted treatment approaches including Onasemnogene Abeparvovec (OA) were introduced. This study describes the first real-world experience with OA in Switzerland.

**Methods:**

Prospective observational case series study using data collected within the Swiss Registry for Neuromuscular Disorders from SMA patients treated with OA. Development of motor, bulbar and respiratory function, appearance of scoliosis, and safety data (platelet count, liver function, and cardiotoxicity) were analyzed.

**Results:**

Nine individuals were treated with OA and followed for 383 ± 126 days: six SMA type 1 (of which two with nusinersen pretreatment), one SMA type 2, and two pre-symptomatic individuals. In SMA type 1, CHOP Intend score increased by 28.1 from a mean score of 20.5 ± 7.6 at baseline. At end of follow-up, 50% of SMA type 1 patients required nutritional support and 17% night-time ventilation; 67% developed scoliosis. The SMA type 2 patient and two pre-symptomatically treated individuals reached maximum CHOP Intend scores. No patient required adaptation of the concomitant prednisolone treatment, although transient decrease of platelet count and increase of transaminases were observed in all patients. Troponin-T was elevated prior to OA treatment in 100% and showed fluctuations in 57% thereafter.

**Conclusions:**

OA is a potent treatment for SMA leading to significant motor function improvements. However, the need for respiratory and especially nutritional support as well as the development of scoliosis must be thoroughly evaluated in SMA type 1 patients even in the short term after OA treatment.

## Background

Chromosome 5q-associated spinal muscular atrophy (SMA) is an autosomal recessive, degenerative neuromuscular disorder characterized by loss of alpha motor neurons in the spinal cord and brainstem. SMA leads to progressive muscle hypotonia, weakness and atrophy, reduced or absent brisk muscle reflexes, tongue fasciculation and respiratory and bulbar impairment [1]. Because of a broad phenotypic spectrum, SMA was classified into subtypes 0 to 4 depending on disease severity, reflected by the age at manifestation and maximum motor functions achieved [2, 3]. The majority of SMA patients has a severe, infantile-onset phenotype (SMA type 1), which leads to early death [4–6]. With an overall incidence of approximately 1 in 6000 to 10,000 live births [7], SMA was a leading cause of infant mortality prior to the introduction of disease modifying therapies. SMA is caused by biallelic mutations of the *survival motor neuron 1* gene (*SMN1*), mapped on chromosome 5q11.1-13.3 and coding for the survival motor neuron protein (SMN) [8]. A homozygous deletion of *SMN1* gene exon 7 (+/- exon 8) can be found in 95% of SMA patients. The remaining cases exhibit either a heterozygous deletion in combination with a sequence variant, or two biallelic sequence variants [9].

Correlation between the clinical severity in SMA and the copy number of the *SMN2* gene, a low-functioning paralogue of *SMN1*, has been described [10]. Therefore, therapies aiming at increasing the SMN protein production by influencing *SMN2* gene splicing have been developed. Treatment with *SMN2* gene splice site modifier can prolong survival and enable the achievement of motor milestones in SMA type 1 patients [11, 12] and preserve motor functions in later onset SMA types 2 and 3 [13, 14]. These new therapies have markedly changed the course of SMA in treated individuals. Meanwhile, two different *SMN2* gene splice site modifiers have been approved for the treatment of SMA in several countries – nusinersen and risdiplam. In Switzerland, nusinersen was approved for the treatment of SMA in September 2017. An evaluation of real-life outcome data of SMA patients treated with nusinersen in Switzerland was published recently using data which was prospectively collected by the Swiss Registry for Neuromuscular Disorders (Swiss-Reg-NMD) [15]. Risdiplam, a different *SMN2* gene splice site modifier, was approved for the treatment of SMA in Switzerland in May 2021. Analysis of the register’s real-world treatment data for risdiplam in Switzerland is not yet available.

A different treatment approach for SMA is the gene addition therapy Onasemnogene Abeparvovec (OA), which was developed in parallel to *SMN2* gene splice site modifier therapies. OA delivers the human *SMN1* gene via an adeno-associated viral vector (serotype 9; AAV9) in a single intravenous dose. OA has demonstrated improved survival and motor milestone achievements for pre-symptomatic individuals and patients with SMA type 1 [16–19]. Recent reviews describe the development of OA and results from clinical trials as well as the already available real-world observations [20, 21]. OA received FDA and EMA approvals for the treatment of SMA in May 2019 and May 2020, respectively. In Switzerland, OA was approved by the regulatory agency (Swissmedic) in July 2021 for the treatment of individuals with 5q-associated SMA below age 2 years and a clinical diagnosis of SMA type 1 or SMA patients with no more than 3 *SMN2* gene copies. Prior to approval, the Swiss Federal Social Insurance Office (FSIO) announced in July 2020 that the Disability Insurance would consider reimbursement for OA treatment of newly diagnosed and treatment-naïve cases. Since September 2020, in total 9 individuals received OA for the treatment of SMA until end of 2021 in Switzerland. This study investigates the real-world practice and describes the outcome with focus on development of motor functions, nutritional and respiratory status, appearance of scoliosis and safety in a first case series of SMA patients treated with OA in Switzerland.

## Methods

This multicenter, prospective observational case series study included data from patients treated with OA in Switzerland in a time period from the early availability of OA (July 2020) until the end of the year 2021. Follow-up data cut off for this study was June 30, 2022.

### Identification of SMA patients and pre-symptomatic SMA individuals, eligibility criteria for OA treatment, OA administration and associated treatment procedures

SMA patients were clinically identified and the clinical suspicion verified by genetic testing. Pre-symptomatic SMA individuals were identified by early postnatal genetic testing which was initiated because of a positive family history for SMA. At the time of this study, SMA was not yet included in the national newborn screening program in Switzerland.

Eligibility criteria for OA treatment were: biallelic *SMN1* gene mutations, maximum of 3 *SMN2* gene copies, age < 2 years, normal range baseline blood values including, but not limited to, platelet count, liver transaminases and anti-AAV9 antibody titers ≤1:50.

All parents provided written informed consent to OA treatment after being informed of the risks and benefits of OA and therapeutic alternatives.

All patients received a single dose of intravenous OA (1.1 × 10^14^ vector genome per kg bodyweight over 60 min) under close monitoring of vital parameters. As recommended, all patients received oral prednisolone 1 mg per kg bodyweight and day for approximately 30 days starting 24 h prior to OA treatment. Prednisolone treatment was then tapered over a period of additional 30 days. Prednisolone dose adjustment and exact treatment duration were on the discretion of the treating physician.

### Functional and laboratory assessments following OA treatment

Post-treatment care including functional and laboratory assessments followed a German consensus paper [22]. Motor function was assessed by trained physiotherapists with standardized clinical assessments which are also used by the global Treat-NMD registry (https://treat-nmd.org), other SMA registries [23–25] and previous clinical trials in SMA [11, 12, 14, 16–19]. For patients < 2 years of age and “non-sitters” the Children’s Hospital of Philadelphia Infant Test of Neuromuscular Disorders (CHOP Intend, total score 0-64) [26–28] and/or the Hammersmith Infant Neurological Examination (HINE, total score 0-26) [29] were used. Depending on individual motor function and/or for older patients the Hammersmith Functional Motor Scale Expanded (HFMSE, total score 0-66) was applied [30–32]. In addition, qualitative motor abilities were recorded for all patients according to the “TREAT-NMD SMA Patient Registry Dataset” (Version 2, https://sma.treat-nmd.org/items/Motor%20function). Laboratory testing included (not limited to) blood cell counts, liver function (aspartate aminotransferase [AST], alanine aminotransferase [ALT], total bilirubin), coagulation profile, and heart function tests (troponin-I and/or troponin-T).

### Data collection and management

Data were prospectively collected before OA treatment and during follow-up according to published recommendations [22]. Data were provided by the treating physicians and collected within the Swiss Registry for Neuromuscular Disorders (Swiss-Reg-NMD; https://www.swiss-reg-nmd.ch). This registry is hosted at the Institute of Social and Preventive Medicine (ISPM), University of Bern, Switzerland, and was approved by the Cantonal Ethics Committee of Bern (20.06.2018, KEK Bern, 2018-00289). Procedures of data collection and processing was described previously [15].

### Statistical analysis

This work is mainly descriptive. If not stated otherwise the mean ± standard deviation is shown.

## Results

### Study participants and OA treatment

Nine individuals were treated with OA in Switzerland between September 2020 and December 2021: six patients with a clinical diagnosis of SMA type 1, one patient with a clinical diagnosis of SMA type 2, and two individuals in a pre-symptomatic disease stage. Two SMA type 1 patients were treated with nusinersen prior to OA administration. All other individuals within this study were nusinersen treatment-naïve. At OA treatment, age of the study population was 160 ± 155 days (range 19-527 days); body weight was 5.9 ± 1.4 (range 4.0-8.7 kg). Follow-up interval after OA treatment was 383 ± 126 days (range 182-621 days). Age at last follow-up for the cohort of clinically diagnosed SMA type 1 patients was 571 ± 175 days (range 374-933 days) and 543 ± 205 days (range 286-933 days) for the entire study case series. For a summary of patient demographics and treatment data please refer to Table 1.

**Table 1:** Summary of patient demographics and treatment data

SMA type	*SMN2* copy number	Nusinersen treatment initiation	Onasemnogene Abeparvovec treatment			last follow-up	
		age [d]	age [d]	body weight [kg]	nutritional and/or ventilatory support; scoliosis	age [d]	nutritional an/or ventilatory support; scoliosis
1b	2	39	102	6,4	–	606	S
1a	2	80	312	7,6	N (gs)	933	N (gs), S
1b	2	–	75	5,3	–	502	S
1b	2	–	97	6,0	–	518	N (gs), S
1b	2	–	79	4,8	–	494	N (ng), V
1b	2	–	192	5,8	–	374	–
2	3 SMN hybrid gene copies	–	527	8,7	–	830	–
ps	3	–	19	4,5	–	286	–
ps	3 SMN hybrid gene copies	–	37	4,0	–	340	–

*ps* pre-symptomatic, *N* nutritional support (gs gastrostomy, ng nasogastric tube), *V* ventilatory support, *S* scoliosis

#### SMA type 1

In two SMA type 1 patients, nusinersen treatment was initiated prior to OA at an age of 39 and 80 days, and discontinued after 3 and 5 doses, respectively. OA was administered at age 102 and 312 days, respectively, in these two patients. The remaining four SMA type 1 patients were nusinersen treatment-naïve and received OA at a mean age of 111 ± 48 days (range 75-192 days). Homozygous *SMN1* gene deletions and two *SMN2* gene copies were found in all six SMA type 1 patients.

#### SMA type 2

One SMA type 2 patient was treated with OA at age 17 months (527 days). This patient harbored 3 copies of a SMN hybrid gene consisting of exons 1-7 of the *SMN2* gene and exon 8 of the *SMN1* gene instead of *SMN1* and *SMN2* genes. Importantly, such a SMN hybrid gene lacks *SMN1* gene exon 7.

#### Pre-symptomatic

Two individuals were treated in a pre-symptomatic disease stage. Of these two individuals, one was identified with a homozygous *SMN1* gene deletion and three *SMN2* gene copies by early postnatal genetic testing because of a positive family history. The other pre-symptomatic individual was 3 weeks old when his older sibling was diagnosed with SMA type 2. Consecutive genetic testing revealed the presence of 3 SMN hybrid gene copies as in the older sibling. OA treatment was initiated at age 19 and 37 days, respectively.

### Overall/event-free survival

None of the SMA patients included in this study died or required permanent ventilation (defined as ≥16 hours/day over ≥14 days) during the follow-up period.

### Motor function

All individuals treated with OA showed clinically meaningful improvements of their motor function as determined by appropriate assessments.

#### SMA type 1

**Fig. 1:**
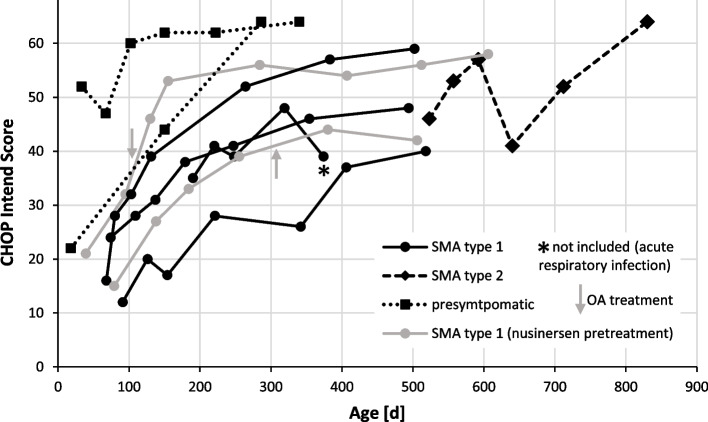
Development of individual CHOP Intend scores of SMA patients and pre-symptomatic individuals following OA treatment. Gray arrows indicate administration of OA in two SMA type 1 patients with nusinersen pretreatment. In all other patients the first assessment was done within 1-7 days prior to OA treatment. Asterix indicates a CHOP Intend score that was not included into the analysis, because the patient was suffering from an acute respiratory infection at the time of the assessment

In SMA type 1 patients, mean baseline CHOP Intend score was 20.5 ± 7.6 (range 12-35) prior to any treatment (including nusinersen pretreatment in two patients) and 26.3 ± 9.9 (12-39) prior to OA treatment, respectively. CHOP Intend score increased to 49.2 ± 7.2 (range 40-59), which corresponds to an increase of mean CHOP Intend score of 28.1 during the observation period following initiation of any disease modifying treatment. Mean observation period until last CHOP Intend assessment was shorter than the entire follow-up period, because in one patient CHOP Intend assessment was replaced by HFMSE and RULM assessments during the follow-up period. Observation period until last CHOP Intend assessment was 406 ± 113 days (range 182-567) after any treatment initiation (including nusinersen pretreatment) and 357 ± 123 days (range 182-504) after OA treatment, respectively. Five SMA type 1 patients became symptomatic and received targeted treatment at age 97 days or younger. One SMA type 1 patient was already 192 days old at treatment start. Individual CHOP Intend baseline scores and score trajectories of the five younger patients demonstrate that scores at baseline and last follow up were higher the younger the patients were at the initiation of treatment (s. Fig. 1).

All SMA type 1 patients but one achieved head control (83%). The patient who did not gain head control had the lowest CHOP Intend score of the entire case series at baseline and last follow-up (12 and 40, respectively). All but one, who was the oldest SMA type 1 patient at treatment start, achieved the ability to roll onto the side. Three patients became sitters (50%). The three sitters at last follow up belonged to the four youngest SMA type 1 patients at treatment initiation (age 39, 75 and 80 days). None of the SMA type 1 patients achieved the ability to crawl, stand or walk with or without support. All SMA type 1 patients developed a useful hand function (100%) and became able to raise their hands to the mouth (100%), but only three patients were able to raise the hands overhead (50%).

#### SMA type 2

One SMA type 2 patient (OA treatment at age 527 days, baseline CHOP Intend score 46) performed heterogeneously in the motor function assessments due to variable cooperation but reached the maximal CHOP Intend score (64) at last follow-up. Assessing motor function with HFMSE, the score increased from 14 at baseline to 25 at last follow-up. During the follow-up period of 303 days this patient gained the ability to stand with assistance.

#### Pre-symptomatic

Two individuals were treated in a pre-symptomatic disease stage. CHOP Intend score before OA treatment was 22 at age 18 days in one (3 *SMN2* gene copies) and 52 at age 37 days in the other individual (3 SMN hybrid gene copies). Following OA treatment CHOP Intend Score increased to the maximum (64) in both, assessed at last follow-up at an age of 286 and 340 days, respectively. Both pre-symptomatically treated individuals showed a normal motor development during the observation period.

Development of CHOP Intend scores in SMA patients treated with OA is illustrated in Fig. 1.

### Nutritional and respiratory support, scoliosis

One SMA type 1 patient, who was pretreated with nusinersen, was dependent on nutritional support with supplementary feeding via nasogastric tube (ng) already prior to OA administration. Gastrostomy (gs) was done 2 weeks before OA administration in this patient. All other study participants did not require nutritional support prior to OA treatment. None of the study participants required respiratory support or had already developed scoliosis prior to OA administration.

At the end of follow-up, 3/6 (50%) SMA type 1 patients required nutritional support via nasogastric tube or gastrostomy. One additional SMA type 1 patient was transiently dependent on supplementary nutritional support via nasogastric tube but fully orally fed at last follow-up. 4/6 (67%) had developed scoliosis (Cobb angle range 20-54°). 1/6 (17%) started night time non-invasive ventilation at age 14 months (11 months after OA treatment) because of sleep disturbed breathing. All other study participants did not require respiratory support at any time of this study. There seemed to be no association between the need of nutritional and respiratory support or the development of scoliosis in SMA type 1 patients and the age at treatment onset or baseline CHOP Intend score: e.g., the individual who required night time ventilation was treated rather early (at age 72 days) and had the second highest baseline CHOP Intend score (24) of the SMA type 1 cohort.

The SMA type 2 patient and the two individuals, who were treated with OA at a pre-symptomatic disease stage, did neither require nutritional or respiratory support nor did they develop a scoliosis until end of follow-up.

### Prednisolone treatment

Prednisolone treatment (1 mg per kg bodyweight and day) was initiated 1 day before OA administration in all patients and continued over a period of 35 ± 9 days (range 23-50), followed by tapering over a period of 34 ± 16 days (range 20-63). No patient required escalation with a higher prednisolone dosage. Duration of full dose prednisolone and tapering was at the discretion of the treating physician and varied rather between the treatment centers than depending on the level of transaminases, e.g.

### Safety measures, post-treatment blood work monitoring

Platelet count decreased in all patients following OA treatment, reaching a nadir of 148 ± 65 G/L (range 36-240) on day 6 ± 2 (range 3-9) after OA treatment. However, thrombocytopenia (defined as platelet count < 150 G/L) occurred only in 3/9 (33%; range 36-123 G/L) on day 6 ± 1 (range 5-7). Both nusinersen pretreated SMA type 1 patients and the oldest/heaviest study participant (SMA type 2) experienced thrombocytopenia. Thrombocytopenia was transient in all patients and did not require any intervention.

Following OA treatment, AST and/or ALT showed fluctuations associated with OA treatment and increased to a first maximum on day 5 ± 1 (range 4-7) in all patients (100%) and to a second peak on day 55 ± 7 (range 43-63) in 4/9 (44%) patients. In two patients, transaminases showed an additional maximum interposed between those two peaks on day 22 and 30 post OA treatment, respectively. At the first peak, AST/ALT levels either remained within the normal range or < 2x ULN in most patients. Only in two patients (one nusinersen pretreated and one treatment naïve SMA type 1 patient), transaminase levels were transiently >2x ULN but < 400 U/L. This transaminase elevation >2x ULN involved both transaminases (AST and ALT) in the nusinersen pretreated patient and only AST in the other patient. The other patient was treated with OA although liver transaminases were mildly elevated (<2x ULN) at baseline. In both cases, transaminase levels returned <2x ULN after 3 days. No adjustment of prednisolone treatment was done in both patients. At the second peak, AST/ALT increase remained within normal range or < 2x ULN in two patients. In two patients, transaminases were transiently >2x ULN, but < 400 U/L. In one of these patients (a nusinersen pretreated SMA type 1 patient, still receiving the full dose of prednisolone), both transaminases AST and ALT were elevated >2x ULN and no adjustment of prednisolone treatment was done. In the other patient (the SMA type 2 patient), only ALT was elevated >2x ULN and prednisolone tapering was slowed in response to the transiently elevated transaminase. Due to longer intervals between lab tests according to the follow-up protocol exact duration of transaminase elevation at the second peak cannot be indicated. In total, in four patients a brief increase in transaminases >2x ULN either at the first or the second peak was observed.

Cardiac surveillance was done investigating Troponin-T in most patients (7/9), because Troponin-I was not routinely available in most Swiss neuromuscular center. Troponin-T was elevated in 7/7 (100%) SMA patients already at baseline (51 ± 25 ng/L; range 22-105). Following OA treatment, Troponin-T levels showed fluctuations which were independent of OA treatment in 3/7. In 4/7 patients, Troponin-T was 41 ± 14 ng/L at baseline and showed an identifiable peak elevation after OA treatment on day 5 ± 2 (range 2-8) and reached 74 ± 13 ng/L (range 52-84). Troponin-I was measured during follow-up in 5/9. Troponin-I was within the normal range at baseline and during follow-up in 4/5. In 1/5 Troponin-I was >ULN at baseline and showed fluctuations unrelated to OA-treatment or other identifiable reasons during follow-up.

### Hospitalizations

According to guidelines and the Swissmedic prescribing information all patients were hospitalized for OA administration. During the follow-up period 5/6 SMA type 1 patients were hospitalized. Four SMA type 1 patients were hospitalized for elective reasons (in total 5 hospitalizations: correction of inguinal hernia, orchidopexy, instruction for nutritional support, gastrostomy placement, initiation of nocturnal non-invasive ventilation). Three SMA type 1 patients required hospital treatment for acute reasons (in total 8 hospitalizations), mainly for the treatment of respiratory tract infections including two hospitalizations because of RSV infections. Although recommended for the non-sitter SMA population during the first 2 years of life, RSV prophylaxis could not be offered to all patients, because reimbursement of Synagis® was denied by some payers due to the lack of approval in Switzerland. None of the study participants required inpatient treatment for COVID-19.

## Discussion

Efficacy and safety data for OA in the treatment of SMA are still limited. This new gene addition therapy has been studied in clinical trials mainly in SMA type 1 patients age < 6 months and pre-symptomatic neonates / young infants age ≤ 6 weeks [16–19]. In several countries and regions OA has been approved for the treatment of SMA and mostly received a much broader approval label than substantiated by scientific evidence from clinical trials. Post-approval use of OA therefore includes patient populations for which in part little efficacy and safety data are available, e.g. SMA patients which are older and/or heavier, have a less severe SMA phenotype and/or were pretreated with other targeted disease modifying therapies. Standardized collection, analysis and publication of treatment and post-treatment surveillance data within specialized disease registries therefore are of utmost importance to document efficacy and safety for a broader patient population, for the long-term evaluation, and for the comparison of the different available disease modifying treatments. Real-world data of OA treatment from registries and individual institutions only start to emerge [33–43]. These publications quite commonly report on small and heterogenous patient cohorts by age, body weight and disease severity, and include a large proportion of nusinersen pretreated patients. The latter takes the historical fact into account that many SMA patients seeking treatment with OA were already receiving a disease modifying therapy that was approved prior to OA, especially nusinersen. Overall, the total number of patients for which OA treatment data are published is still very low compared to the total number of patients treated with OA, which is > 2300 as of August 2022 according to the OA manufacturer’s self-declaration [44]. This study uses data which was prospectively collected in the Swiss Registry for Neuromuscular Disorders (Swiss-Reg-NMD) and aims at adding treatment data to the dataset of worldwide real-world observations from the first SMA patients treated with OA in Switzerland.

Our study case series reflects the approval and the reimbursement regulations in Switzerland. OA received Swissmedic approval in July 2021 for the treatment of individuals with 5q-associated SMA below age 2 years and a clinical diagnosis of SMA type 1 or SMA patients with no more than three SMN2 gene copies (https://www.swissmedic.ch/swissmedic/de/home/humanarzneimittel/authorisations/new-medicines/zolgensma-infusionsloesung-onasemnogen-abeparvovec.html). Prior to approval, the responsible payer (Disability Insurance) considered reimbursement of OA on a case-by-case basis since July 2020. This decision process followed internal regulations of the Disability Insurance that were already defined with the market introduction of nusinersen (Spinraza®) in Switzerland and exclude the reimbursement of OA when an individual SMA patient already receives treatment with an *SMN2* gene splice site modifier. Therefore, the proportion of SMA patients with nusinersen pretreatment in our case series is low when compared to other real-world studies from other countries and regions [33–43]. Only two out of nine study participants (both SMA type 1) received nusinersen treatment prior to OA. One patient (previously treated with five doses of nusinersen) covered the OA treatment expenses by private funds and was, therefore, not dependent on reimbursement regulations. The other patient was treated with nusinersen immediately after the SMA diagnosis based on the joint decision of the treating physician and the parents as the patient had an active respiratory infection. Parents however questioned their decision after initiation of nusinersen treatment even though the patient had gained in motor function, and asked for OA treatment. Following intensive discussion, the payer exceptionally agreed to reimburse OA although this patient has been pretreated already with 3 doses of nusinersen. This case reflects the fact that decision making for the treatment of a life-threatening condition is extremely demanding, especially when decisions have to be made under time pressure and in an emotional state of exception, when disease and treatment mechanisms are complex and difficult for laypersons to comprehend, when little is known about differences between available targeted SMA therapies regarding long term efficacy and superiority / inferiority of available treatment options, and when treatment related expenses are extreme and therefore may be limited by regulations of the payers.

Interestingly, two individuals (siblings) in this case series harbored three copies of a hybrid SMN gene. This hybrid SMN gene consists of exon 1-7 of the centromeric *SMN2* gene and exon 8 of the telomeric *SMN1* gene. Such a hybrid SMN gene lacks *SMN1* gene exon 7 and, therefore, causes SMA. Hybridization of *SMN1* and *SMN2* genes was described already shortly after *SMN1* gene identification [8, 45] and was proven recently as a relatively frequent variation in *SMN2* gene copies in SMA patients [46].

Selection of study populations in clinical trials may only reflect a proportion of the disease spectrum of a certain condition. Expectations on treatment efficacy based on a clinical trial may therefore be valid only for a distinct study population. This is also true for the OA trial program – a fact, which emphasizes the importance of collecting and analyzing real-world data. For example, mean CHOP Intend scores at baseline were 32.0 ± 9.7 and 27.9 ± 8.3 in the phase 3 STR1VE-US and STR1VE-EU trials [16, 17], whereas natural history studies of SMA type 1 document a mean CHOP intend score at baseline between 20.2 ± 7.4 and 27.4 ± 8.5 [47, 48]. Considering that a difference of four points on the CHOP intend scale is clinically significant, and that one conclusion from the OA phase 1 trial START and its long-term follow-up study is that the individual outcome following OA treatment might be better with a higher baseline motor function score [49], differences in baseline motor function between trial and real-world data might explain differences between the expected (based on trial results) and real-world outcome. In our case series, the mean baseline CHOP intend score of SMA type 1 patients was 20.5 ± 7.6 and compared to natural history studies [47, 48] rather on the lower end of the mean baseline motor function in the SMA type 1 population. Thus, although considerably lower than in the clinical trials [16, 17] our patient case series showed a rapid and sustained mean increase of 28.1 in total CHOP Intend score from baseline during the observation period and achievement of motor milestones, that were comparable to results from clinical trials, e.g. independent sitting in 50%. However, the need for nutritional and respiratory support and the prevalence of scoliosis in our case series at last follow-up was higher than expected based on trial results. Whether this might be explained by the lower level of motor function of our small case series at baseline, the selection bias of clinical trial populations or individual disease trajectories of our study population can only be speculated.

Post-treatment blood work monitoring in our case series showed previously described hematologic abnormalities (decreased platelet counts and thrombocytopenia) and signs of hepatotoxicity (increased levels of transaminases) following OA administration. In our case series, these laboratory findings were transient and did not require adaptations of the concomitant steroid treatment or other measures. Some adverse events may be associated with the total viral vector dose (and, thus, body weight) and a potential pretreatment with nusinersen prior to OA administration as observed and discussed by some of the early reports from real-world data [36, 38]. Also in our case series, the most pronounced abnormalities in platelet counts and transaminase levels were observed in the oldest (= heaviest) study participant, the SMA type 2 patient, and the two nusinersen pretreated SMA type 1 patients. However, it is important to mention that adverse events after OA treatment can be severe with even fatal outcome in rare cases due to thrombotic microangiopathy [50] and hepatic failure [44].

The new targeted treatment options including OA have been proven to be potent disease modifying therapies in the treatment of SMA in symptomatic patients. When treatment is initiated in a pre-symptomatic disease state within the first weeks of life, however, motor function in SMA may even occur within the expected normal range, at least in early life [18, 19, 51]. This ultimately leads to a significant reduction of the burden of disease, both for the individual patient and for the society. Early identification of the condition can be achieved by newborn screening (NBS). SMA, therefore, was included into many national newborn screening (NBS) programs worldwide. Application for the inclusion of SMA into the Swiss NBS program is about to be filed at the time of submission of this report.

## Conclusion

This study describes the first experience with OA treatment in Switzerland. Our case series study demonstrates that OA is an effective disease modifying treatment for SMA, a result that also was shown in clinical trials and other real-world studies. All study participant showed significant improvements of their motor function. However, the need for respiratory and especially nutritional support as well as scoliosis development was common in SMA type 1 patient in our case series and must be thoroughly evaluated even in the short term after OA treatment.

## Data Availability

The datasets generated and analyzed during the current study are not publicly available because they would allow identification of individual patients even after anonymization. However, the datasets are available from the corresponding author on reasonable request.
